# Morphological evaluation of gonial and antegonial regions in bruxers on panoramic radiographic images

**DOI:** 10.1186/s12903-023-03162-0

**Published:** 2023-07-07

**Authors:** Zerrin Unal Erzurumlu, Elif Sadik, Busra Nur Gokkurt, Furkan Ozbey, Kerem Erzurumlu, Yeliz Kasko Arici

**Affiliations:** 1 Department of Dentomaxillofacial Radiology, Faculty of Dentistry, Ordu University, Ordu, 52200 Turkey; 2 Computer Science and Engineer, Department of Computer Programming, Vocational School of Technical Sciences, Ordu University, Ordu, Turkey; 3 Department of Biostatistics and Medical Informatics, Faculty of Medicine, Ordu University, Ordu, Turkey

**Keywords:** Bruxism, Cortical bone, Fractal Analysis, Mandible, Trabecular bone

## Abstract

**Background:**

This is the first study to report both cortical and trabecular bone evaluation of mandibles in bruxers, within the knowledge of the authors. The purpose of this study was to evaluate the effects of bruxism on both the cortical and the trabecular bone in antegonial and gonial regions of the mandible, which is the attachment of the masticatory muscles, by using panoramic radiographic images.

**Methods:**

In this study, the data of 65 bruxer (31 female, 34 male) and 71 non-bruxer (37 female, 34 male) young adult patients (20–30 years) were evaluated. Antegonial Notch Depth (AND), Antegonial-Index (AI), Gonial-Index, Fractal Dimension (FD) and Bone Peaks (BP) were evaluated on panoramic radiographic images. The effects of the bruxism, gender and side factors were investigated according to these findings. The statistical significance level was set at*P* ≤ 0.05.

**Results:**

The mean AND of bruxers (2.03 ± 0.91) was significantly higher than non-bruxers (1.57 ± 0.71; *P* < 0.001). The mean AND of males was significantly higher than females on both sides (*P* < 0.05). The mean AI of bruxers (2.95 ± 0.50) was significantly higher than non-bruxers (2.77 ± 0.43; *P* = 0.019). The mean FD on each side was significantly lower in bruxers than in non-bruxers (*P* < 0.05). The mean FD of males (1.39 ± 0.06) was significantly higher than females (1.37 ± 0.06; *P* = 0.049). BP were observed in 72.5% of bruxers and 27.5% of non-bruxers. The probability of existing BP, in bruxers was approximately 3.4 times higher than in non-bruxers (*P* = 0.003), in males was approximately 5.5 times higher than in females (*P* < 0.001).

**Conclusion:**

According to the findings of this study, the morphological differences seen in cortical and trabecular bone in the antegonial and gonial regions of the mandible in bruxers can be emphasized as deeper AND, higher AI, increased of existing BPs, and lower FD, respectively. The appearance of these morphological changes on radiographs may be useful for indication and follow-up of bruxism. Gender is an effective factor on AND, existing BP and FD.

## Background

Bruxism is a common parafunctional habit characterized by clenching and/or grinding [1]. Bruxism can occur during sleep (nighttime bruxism/sleep bruxism/nocturnal bruxism) or awake (daytime bruxism/wakefulness bruxism/diurnal bruxism) [2]. Sleep bruxism affects approximately 80–95% of the world’s population and is more common in individuals aged 15–40 [3]. It is a clinically important phenomenon as it is commonly encountered in the adult population [1, 4]. Factors such as alcohol, tobacco, medicine, oral habits, temporomandibular joint disorders, malocclusion, hypopnea, high anxiety level, psychiatric disorders and stress can affect the formation of bruxism [1]. Bruxism; can result in periodontal loss and mobility of teeth, tooth wear and fractures, temporomandibular joint dysfunction, and pain in the chewing system and orofacial structures [2].

The bones in the maxillofacial region are under forces due to the contraction and movement of attached human masticatory muscles, which are largely specialized in jaw movement [5, 6]. These forces can cause morphological changes on the mandible [5]. Many studies suggest that changes in masticatory muscle activity lead to changes in mandible morphology [7–10]. Gonial and antegonial regions are the areas of remodeling in the mandible, such as ramus and condyle [11]. The gonial region is the junction of the mandibular body and ramus. The antegonial notch region is defined as the upward curve of the lower border of the mandible in front of the gonion [12].

Bruxism can influence the remodeling of the gonial and antegonial region, which is a unit of remodeling. Attachment of medial pterygoid muscles and masseter to the mandibular gonial and antegonial regions may cause this region to be more affected by parafunctional conditions [13, 14]. Studies examining the mandible in patients with bruxism were published in the literature [2, 4, 15, 16]. However, in the literature there is no study that assesses specifically both the cortical and the trabecular bone of antegonial and gonial regions of the mandible in bruxers. Therefore, the objectives were: (1) To evaluate the effects of bruxism on both the cortical and the trabecular bone in antegonial and gonial regions of the mandible, which is the attachment of the masticatory muscles, by using panoramic radiographic images. (2) The null hypothesis of this study is that cortical and the trabecular bone in the mandibular antegonial and gonial regions are not different between bruxers and non-bruxers. (3) The specific aim of this study was to compare the antegonial and gonial regions on panoramic radiographic images in bruxers and non-bruxers by evaluating Antegonial Notch Depth (AND), Antegonial-Index (AI), Gonial-Index (GI), existence of Bone Peaks (BP) for cortical bone, and Fractal Dimension (FD) for trabecular bone.

## Materials and methods

### Study design and sample

Bruxer and non-bruxer young adults (20–30 years) were admitted to Ordu University Faculty of Dentistry Department of Oral and Maxillofacial Radiology for routine examination. Patients, who did not have any systemic disease related to bone metabolism or did not have any systemic disease which may result in increased liability to chewing disorder, were included in the study. Patients were evaluated as bruxer if they have a history of clenching or grinding during the day or night, if a grinding sound was reported by a partner while sleeping, and if they experience tension, pain and fatigue in the masticatory muscles (temporal and/or masseter) after waking up or during the day [2, 4]. Patients who did not have a bruxer history were considered as non-bruxer. Patients were excluded if they were missing teeth except the third molar, had orthodontic treatment, were receiving orthodontic treatment, had pathology in the maxillofacial region, any prosthetic restoration, alcohol and drug addiction, neurological and psychiatric diseases. An ethical approval was obtained from Ordu University Clinical Research Ethics Committee (2023/42) and informed consent was obtained from patients who agreed to participate.

A priori power analysis using the G*power software (version 3.1.9.7) was performed to detect a sample size. The power analysis showed that a total of 128 observations (64 in each group) were required to demonstrate a clinically meaningful difference between the two groups at a 2-sided significance level of 0.05 and 80% power using a Student t-test; based on moderate effect size (Cohen’s d [d] = 0.50).

### Radiographic analysis

All panoramic radiographic images were acquired using the Planmeca Promax 2D (Planmeca Inc., Helsinki, Finland) digital panoramic X-ray device. Images were taken in the standardized position with the parameters 65 kV, 5 mA and 14 s, following the manufacturer’s recommendations.

### Study variables and data collectıon

AND, AI, GI and BP evaluations were made using Turcasoft software (Turcasoft Dent, Samsun, Turkey). The calibration of the measurements is done via Turcasoft’s calibrate option. The observers used HP (Hewlett-Packard, Palo Alto, CA, USA) brand ProDisplay P201 model 20” led monitor with 1600 × 900 resolution in a dimmed environment from a distance of 40 cm. All measurements on panoramic radiographic images were made by the same observer twice with 30 days intervals. BP were evaluated by two observers two times with 15 days intervals.

**Fig. 1a:**
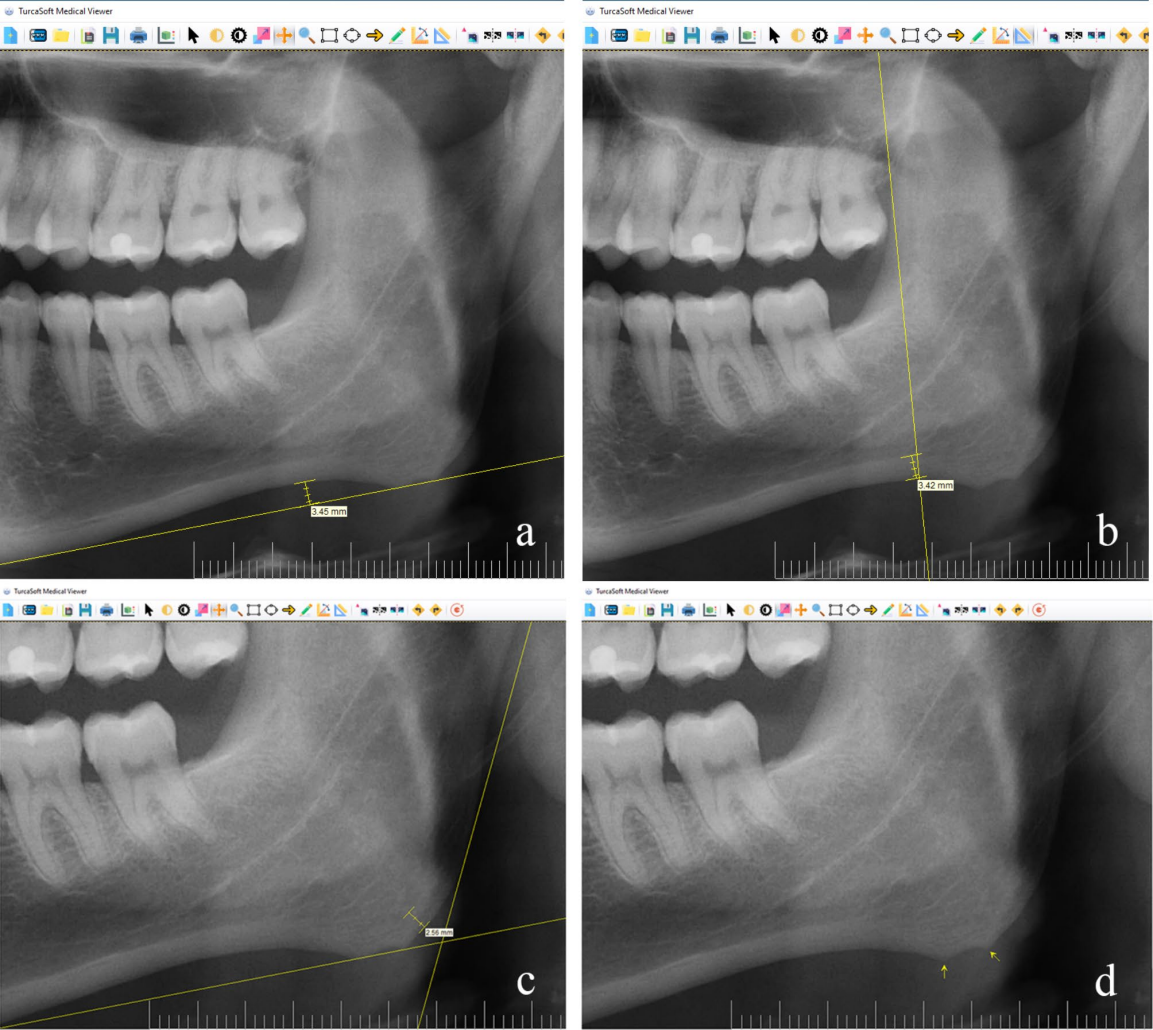
Antegonial notch depth **b.** Antegonial index and **c.** Gonial index measurement, and **d.** Bone peaks evaluation

AND was measured by measuring the perpendicular distance between the tangent line drawn to the lower border of the mandible and the deepest point of the antegonial region [11] (Fig. 1a).

For the AI, cortical bone thickness was measured at the point where the best straight line drawn along the anterior edge of the ascending ramus intersects the inferior border of the mandible [17] (Fig. 1b).

Cortical bone thickness at the gonial angle (the angle between the tangent line to the lower border of the mandible and the tangent line to the posterior border of the ramus) was measured for the GI [17] (Fig. 1c).

Bone appositions in the cortex between the antegonial notch and the gonial angle were evaluated as BP (Fig. 1d). If BP was present on the right and/or left side, it was considered as positive (+); absence on both sides was considered as negative (-).

**Fig. 2:**
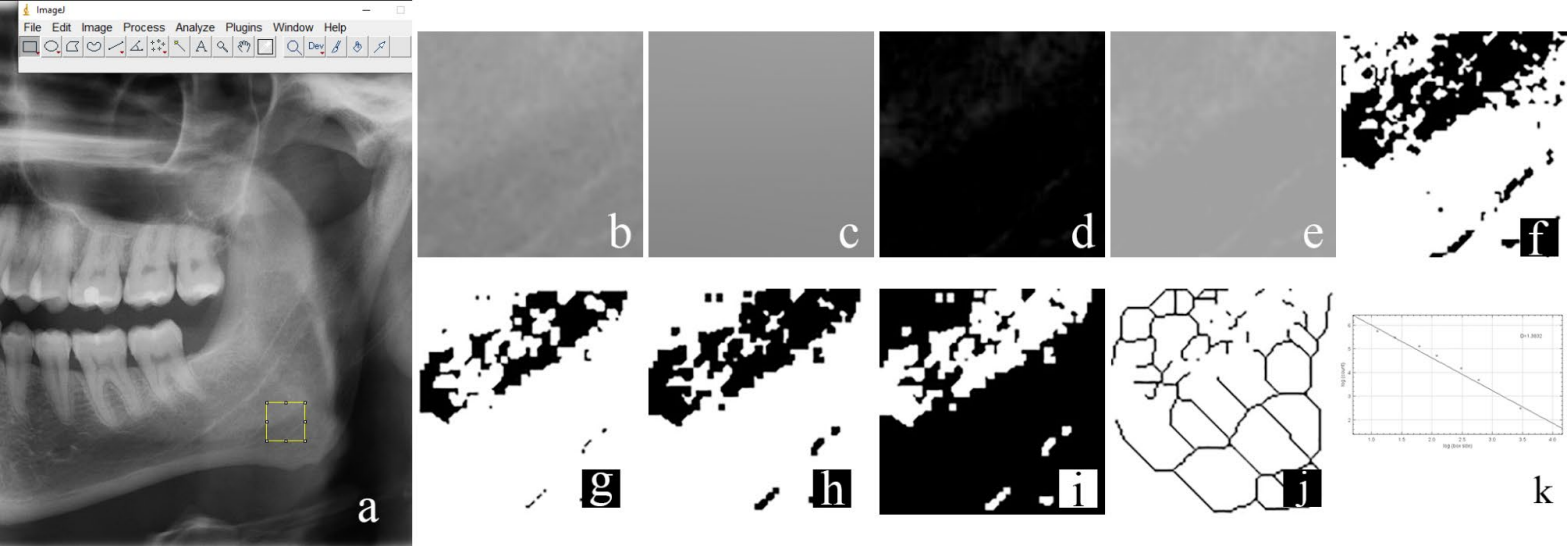
Measurement of fractal dimension, **(a)** Selection, **(b)** Duplication, **(c)** Blurring, **(d)** Subtraction, **(e)** Addition of 128, **(f)** Binary, **(g)** Erode, **(h)** Dilate, **(i)** Invert, **(j)** Skeletonize, **(k)** The box counting procedure

For fractal analysis, a region of interest (ROI) with a size of 100 × 100 pixels on both side in the gonial region was selected on panoramic radiographic images with 300 dpi tagged image file format (TIFF), by a dentomaxillofacial radiologist using the ImageJ software (ImageJ 1.52a, National Institute of Health, Maryland, United States) (Fig. 2a). White and Rudolph’s [18] method was used for measurement of FD. The method of White and Rudolph [18] includes a series of processing steps. First, the duplicated ROI (Fig. 2b) is blurred (“Gaussian Blur”, sigma = 35) (Fig. 2c). The blurred images are then subtracted from the original image and a value of 128 Gy is added for each pixel (Fig. 2d-e). Then the image is converted to a black and white bicolor format by using the “Make Binary” option (Fig. 2f). Then the “Erode” option is applied to reduce the noise in the image (Fig. 2g). Then, with the “Dilate” option, the existing areas are enlarged (Fig. 2h) and made more prominent. In the “Invert” step, the borders of the trabecular bone are revealed by changing the black and white pixels (Fig. 2i). Finally, by using the “Skeletonize” option, the image with trabecular structure is converted into a skeleton structure format and made ready for fractal analysis (Fig. 2j). To calculate the FD, “Fractal Box Counter” option under the “Analyze” tab is selected (Fig. 2k). These processing steps were automated by a computer engineer with the created interface in the ImageJ software. The FD of each selected ROI was calculated using this new interface.

### Statistical analysis

The normality of data was tested using Shapiro-Wilks test and found to be normally distributed (*P* > 0.05). The homogeneity of group variances was tested using Levene’s test. Comparing bruxism groups, a three-way repeated-measure ANOVA main factors were used: side(within-subject), gender(between-subject), and bruxism(between-subject). Univariate binary logistic regression was used to calculate Odds Ratios (OR) and 95% Confidence Intervals (CI) between BP positivity and bruxism, and gender. Qualitative data were presented as frequencies and percentages, and Chi-square test was used in the analysis. Intraclass correlation coefficient (ICC) based on a single-measurement, consistency-agreement, two-way mixed effects model was calculated to measure the intra-rater agreement for the quantitative measurements. Cohen’s kappa coefficient was calculated to measure the inter-rater and intra-rater agreement for the qualitative data. The statistical significance level was set at *P* ≤ 0.05. IBM SPSS 28(IBM Ltd., Tokyo, Japan) was used as statistical software.

## Results

The sample of the study consisted of 136 patients, 50% (n = 68) female and 50% (n = 68) male. In this study, the data of 65 bruxers (31 female, 34 male) and 71 non-bruxers (37 female, 34 male) patients were evaluated. The mean age of the patients was 23.21 ± 2.48 (20–30) years. There was no significant difference between the mean age of bruxer (23.51 ± 2.77) and the mean age of non-bruxer (22.89 ± 2.09) (*P* = 0.397).

ANOVA for AND measurements reported that the Side×Gender interaction was significant (*P* = 0.041). Accordingly, the Bonferroni post hoc test results are shown in Table 1 with letters. On both sides the mean AND of males was higher than females (*P* < 0.05). While there was a significant difference between the mean of the right and left side in males (*P* < 0.05), there was no significant difference in females (*P* > 0.05). The mean AND of bruxers was significantly higher than non-bruxers (*P* < 0.001).

**Table 1:** Descriptives statistics of antegonial notch depth

Side		Gender	Non-bruxers			Bruxers			Total		
			n	Mean	SD	n	Mean	SD	n	Mean	SD
Right		Male	34	1.82	0.54	34	2.55	1.03	68	2.19^**Aa**^	0.90
		Female	31	1.39	0.83	37	1.62	0.72	68	1.51^**Ba**^	0.77
		Total	65	1.62	0.72	71	2.07	0.99			
Left		Male	34	1.69	0.59	34	2.36	0.81	68	2.021^**Ab**^	0.78
		Female	31	1.36	0.76	37	1.64	0.69	68	1.51^**Ba**^	0.73
		Total	65	1.53	0.69	71	1.99	0.83			
*Total*			*130*	*1.57*	*0.71*	*142*	*2.03*	*0.91*			
P*	Side: 0.033; Gender: <0.001; **Bruxism: <0.001** **Side×Gender Int: 0.041**; Side×Bruxism Int: 0.987; Gender×Bruxism Int: 0.077 Side×Gender×Bruxism Int: 0.436										

SD: Standard Deviation

Int: Interaction

*: Comparing bruxers and non-bruxers, a three-way repeated-measure ANOVA was used and main factors were: side (within-subject), gender (between-subject), and bruxism (between-subject). The statistical significance level was set at *P* ≤ 0.05

On the same side, the genders that don’t share a common capital letter are significantly different (*P* < 0.05)

In the same gender, the sides that don’t share a common small letter are significantly different (*P* < 0.05)

In Table 2, ANOVA for AI measures reported significant differences only between the bruxers and non-bruxers. The mean AI of bruxers was significantly higher than non-bruxers (*P* = 0.019). The interactions between the factors were not significant (*P* > 0.05).

**Table 2:** Descriptives statistics of antegonial-index

Side		Gender	Non-bruxers			Bruxers			Total		
			n	Mean	SD	n	Mean	SD	n	Mean	SD
Right		Male	34	2.92	0.36	34	2.96	0.52	68	2.94	0.45
		Female	31	2.69	0.35	37	2.95	0.45	68	2.83	0.42
		Total	65	2.81	0.37	71	2.95	0.48			
Left		Male	34	2.78	0.41	34	2.98	0.56	68	2.88	0.50
		Female	31	2.69	0.54	37	2.91	0.49	68	2.81	0.52
		Total	65	2.73	0.47	71	2.94	0.52			
*Total*			*130*	*2.77*	*0.43*	*142*	*2.95*	*0.50*			
P*	Side: 0.118; Gender: 0.195; **Bruxism: 0.019** Side×Gender Int: 0.540; Side×Bruxism Int: 0.214; Gender×Bruxism Int: 0.425 Side×Gender×Bruxism Int: 0.054										

SD: Standard Deviation

Int: Interaction

*: Comparing bruxers and non-bruxers, a three-way repeated-measure ANOVA was used and main factors were: side (within-subject), gender (between-subject), and bruxism (between-subject). The statistical significance level was set at *P* ≤ 0.05

In Table 3, no statistically significant difference was found in the result of ANOVA for GI measurements (*P* > 0.05). Bruxers’ mean GI was not different from non-bruxers (*P* = 0.066).

**Table 3:** Descriptives statistics of gonial-index

Side		Gender	Non-bruxers			Bruxers			Total		
			n	Mean	SD	n	Mean	SD	n	Mean	SD
Right		Male	34	1.21	0.20	34	1.32	0.31	68	1.27	0.27
		Female	31	1.15	0.30	37	1.25	0.30	68	1.21	0.30
		Total	65	1.18	0.25	71	1.29	0.31			
Left		Male	34	1.25	0.22	34	1.33	0.37	68	1.29	0.30
		Female	31	1.21	0.34	37	1.26	0.23	68	1.23	0.28
		Total	65	1.23	0.28	71	1.29	0.30			
*Total*			*130*	*1.21*	*0.27*	*142*	*1.29*	*0.30*			
P*	Side: 0.225; Gender: 0.180; Bruxism: 0.066 Side×Gender Int: 0.905; Side×Bruxism Int: 0.300; Gender×Bruxism Int: 0.830 Side×Gender×Bruxism Int: 0.880										

SD: Standard Deviation

Int: Interaction

*: Comparing bruxers and non-bruxers, a three-way repeated-measure ANOVA was used and main factors were: side (within-subject), gender (between-subject), and bruxism (between-subject). The statistical significance level was set at *P* ≤ 0.05

BP were observed in 72.5% of bruxers and 27.5% of non-bruxers. It was determined that there was a significant relationship between the intervals (*P* = 0.002). The BP ratio was approximately 3 times higher in patients with bruxism (Table 4).

**Table 4:** The relationship between bruxism and bone peaks

Bruxism	Bone peaks				*Total*		*P*
	Negative (-)		Positive (+)				
	n	%	n	%	*n*	*%*	
No	54	*56.3*	11	27.5	*65*	*47.8*	**0.002** ^**a**^
Yes	42	43.8	29	72.5	*71*	*52.2*	
*Total*	*96*	*100.0*	*40*	*100.0*	*136*		

Qualitative data were presented as frequencies and percentages

^a^: Pearson Chi-Square test

The results of binary logistic regression analysis for BP positivity have been shown in Table 5. As a result of binary logistic regression analysis, both bruxism and gender were determined as effective factors on BP positivity. The probability of existing BP, in bruxers was approximately 3.4 times higher than in non-bruxers (OR, 3.390; 95%CI, 1.519–7.564), in males was approximately 5.5 times higher than in females (OR, 5.492; 95%CI, 2.351–12.829).

**Table 5:** Results of binary logistic regression analysis for Bone peaks positivity

	b	SE	Wald	df	*P*	OR (95% CI)
Bruxism (Yes)	1.221	0.410	8.885	1	**0.003**	3.390 (1.519–7.564)
Gender (Male)	1.703	0.433	15.487	1	**< 0.001**	5.492 (2.351–12.829)

b: Regression coefficient

SE: Standard Error

df: Degrees of freedom

OR: Odds Ratio

CI: Confidence Interval

In Table 6, ANOVA for fractal measurements reported that the Side×Bruxism interaction was significant (*P* = 0.027). Bonferroni post hoc test results are shown in Table 6 with letters. The measured mean FD on each side was significantly lower in bruxers than in non-bruxers (*P* < 0.05). There was no significant difference between the right and the left FDs in both non-bruxers and bruxers (*P* > 0.05). The mean FD of males was significantly higher than females (*P* = 0.049).

**Table 6:** Descriptives statistics of fractal dimension

Side		Gender	Non-bruxers			Bruxers			Total		
			n	Mean	SD	n	Mean	SD	n	Mean	SD
Right		Male	34	1.40	0.06	34	1.38	0.06	68	1.39	0.06
		Female	31	1.39	0.06	37	1.36	0.06	68	1.37	0.06
		Total	65	1.39^**Aa**^	0.06	71	1.37^**Ba**^	0.06			
Left		Male	34	1.41	0.05	34	1.38	0.07	68	1.39	0.06
		Female	31	1.39	0.06	37	1.35	0.06	68	1.37	0.06
		Total	65	1.40^**Aa**^	0.05	71	1.36^**Ba**^	0.06			
*Total*			*130*	*1.40*	*0.06*	*142*	*1.37*	*0.06*			
P*	Side: 0.586; **Gender: 0.049**; Bruxism: 0.010 Side×Gender Int: 0.392; **Side×Bruxism Int: 0.027**; Gender×Bruxism Int: 0.624 Side×Gender×Bruxism Int: 0.889										

SD: Standard Deviation

Int: Interaction

*: Comparing bruxers and non-bruxers, a three-way repeated-measure ANOVA was used and main factors were: side (within-subject), gender (between-subject), and bruxism (between-subject). The statistical significance level was set at *P* ≤ 0.05

On the same side, the bruxism groups that don’t share a common capital letter are significantly different (*P* < 0.05)

In the same bruxism group, the sides that don’t share a common small letter are significantly different (*P* < 0.05)

The calculated ICC values and 95% CI for intra-rater agreement in measures of the AND, AI, GI and FD were found as 0.998, 0.975, 0.847 and 0.818, respectively. Calculated Cohen’s kappa coefficients for inter-rater and intra-rater agreement in measures of the BP diagnosis was found to be between 0.826 and 0.975.

## Discussion

Within the knowledge of the authors, this is the first study to report both cortical and trabecular bone evaluation in the antegonial and gonial regions of the mandible in a group of populations with and without bruxism. According to the findings of this study, AND was deeper, AI was higher, the frequency of BP was higher, and FD was lower in the antegonial and gonial regions in bruxers. Thus, the null hypothesis was rejected for AND, AI, BP and FD, accepted for GI.

In this study, we compared the gonial and antegonial region morphology in bruxer and non-bruxer with the measurements we made on panoramic radiographic images. Panoramic radiography is an imaging technique that shows both dental arches and related anatomical structures on a single film with a simple and rapid procedure. Panoramic radiography is widely used in dental practice as it allows easy examination of the maxillary and mandibular arches, alveolar bone, temporomandibular joints, and adjacent structures [19]. Anomalies such as alveolar bone resorption, decreased mandibular cortical thickness and osteoporosis can also be observed on panoramic radiographic images [20]. The quality and quantity of bone can be determined on panoramic radiographic images with radiomorphometric measurements. These measurements allow the radiographic evaluation of mandibular bone changes [21]. The use of panoramic radiographic images for measurements is controversial due to considerations of magnification and distortion. On the other hand, studies suggest that if the patient is accurately positioned, vertical and angular measurements may be accurately performed. In addition, calibration can be used for 1:1 image virtualization on measurements which is taken from the screen with the help of magnification in digital panoramic radiographs. Some researchers have concluded that vertical measurements taken on panoramic radiographs are acceptable when a calibrated measuring instrument is used [22–24].

Bone structure is affected by physical and chemical stimuli such as function, age, gender, hormones and drugs [25, 26]. Bone mass increases from infancy to approximately 30 years of age, and it then begins to decrease in stages [27]. For this reason, the data of young adults were evaluated in the current study in which we examined the effects of bruxism on the mandible.

In this study the mean AND of bruxers was significantly higher than non-bruxers (*P* < 0.001). Isman [4] also found that the mean AND value was significantly higher in bruxers compared to non-bruxers in her study. In the literature, there are studies reporting that edentulous individuals, who are thought to have lower bite force, have higher AND than dentulous and partially dentulous individuals [12, 28]. It has been reported that this may result from the destructive effects of both increased and decreased bite force [8]. In this study, the AND values of males were significantly higher than females on both sides (*P* < 0.05). The findings of the current study are consistent with the literature [11, 12, 28]. In studies reporting the relationship between the gender and AND, it has been reported that AND is higher in males than in females [11, 12, 28] and emphasized that this situation can be used in forensic dentistry [12]. In the current study, side and gender interaction was found to be significant for AND (*P* = 0.041). Gosh et al. [11] stated that there was no significant difference between the right and left sides of both genders in the 20–29 age group. In the literature the studies that found a significant difference in terms of AND between the right and left sides, the right side AND was reported to be higher than the left side [12, 29]. In this study, the right side AND was higher than the left side in males. The results of the current study are partially compatible with the study of Gosh et al. [11], Ledgerton et al. [17] and Preston et al. [29].

AI and GI are showing the mandibular cortical bone thickness, and were used to measure and evaluate the bone quality and quantity of the mandible [17, 30, 31]. Isman [4] stated that they found the mean GI values to be significantly higher in male bruxers, but there was no difference in terms of AI. In this study, the mean AI value was found to be significantly higher in bruxers than in non-bruxers (*P* = 0.019). Although the mean GI value was higher in bruxers than in non-bruxers, the difference was not statistically significant (*P* = 0.066). In previous studies on long bones, it has been reported that periosteum and periosteal bone apposition cause local cortical thickening due to vascularization and tension caused by muscles [32]. It can be thought that cortical thickening occurs as a reactive response of the mandibular cortex as a result of the pressure exerted on the mandibular corpus by the excessive bite force due to bruxism [33]. It can be argued that cortical thickness in both antegonion and gonion may be affected by local effects of muscle attachments. However, it is emphasized that AI is more consistent than GI because it is thicker, easier to see and sharper [17]. In this study, intra-rater agreement was also found to be higher in AI than GI. In the current study, there was no difference between the genders in terms of both mean AI and GI (0.195 and 0.180, respectively). Isman [4] also stated in her study that there was no correlation between gender and AI in both bruxers and non-bruxers. Palaskar et al. [34] reported that there was no significant difference between the genders in terms of GI. The results of the current study are compatible with the literature.

As muscle strength increases, bone mineralization increases proportionally [35]. Based on this relationship between the bone and the muscle, appositional changes were observed in the mandibular gonial region as a functional adaptation in bruxers [4, 36–38]. Isman [4] reported a significantly higher rate of bony apposition called tiny BP at the cortex of the mandibular gonial region in bruxers than in non-bruxers. Casazza et al. [38] reported that the frequency of BP on the right and left sides was higher in bruxers (83% and 81%, respectively) than in non-bruxers (25% and 17%, respectively). In this study, these frequencies were found to be 72.5% in bruxers and 27.5% in non-bruxers. Turp et al. [36] suggested that radiologically diagnosed appositional changes at the mandibular angle may be an indication or confirmation of bruxism. A significant relationship was found between bruxism and BP in the current study (*P* = 0.002). The rate of BP in bruxers was approximately 3 times higher than in non-bruxers. The probability of existing BP, in bruxers was approximately 3.4 times higher than in non-bruxers (*P* = 0.003), in males was approximately 5.5 times higher than in females (*P* < 0.001).

Fractal analysis is a method that displays the degree of complexity in shapes and structures as a numerical value called FD. Structures with a high FD value are considered more complex, while structures with a lower FD value are considered to have a simpler internal layout [39]. Attachment of the masseter and medial pterygoid muscles to the mandible in the gonial region may cause this region to be more affected by parafunctional conditions [13, 14]. Eninac et al. [15] reported that the FD in the gonial region was significantly lower in bruxers compared to non-bruxers. The findings of the current study are consistent with the studies that found a lower FD in the gonial region in bruxers [2, 15, 40]. In this study, consistent with the other studies, no significant difference was found between the right and left side FDs [15, 40]. Consistent with the literature that suggests gender has an effect on trabecular structure and FD [2, 41, 42], in the current study the mean FD of males was found to be significantly higher than females (*P* = 0.049). This finding may be due to sex-related differences in muscle strength and hormonal and metabolic variations.

The limitations of this study are that it is not known how long the individuals included in the study have been bruxer, polysomnography was not used in the diagnosis of bruxism, the severity of bruxism was not measured, the chewing side preference, if any, was not recorded, the chewing muscle thickness and bite force were not evaluated. Other limitations are that we did not exclude the patients with impacted teeth and with oral habits as tongue trust, which may affect bone morphology, as noted in previous studies [6, 43].

## Conclusion

In conclusion, according to the findings of this study, the morphological differences seen in cortical and trabecular bone in the antegonial and gonial regions of the mandible in bruxers can be emphasized as deeper AND, higher AI, increased of existing BPs, and lower FD, respectively. The appearance of these morphological changes on radiographs may be useful for indication and follow-up of bruxism. Gender is an effective factor on AND, existing BP and FD. Further studies are needed with specific methods used in the diagnosis of bruxism.

## Data Availability

The data that support the findings of this study are available from the corresponding author upon reasonable request.
